# Do Adolescent Idiopathic Scoliosis (AIS) Neglect Proprioceptive Information in Sensory Integration of Postural Control?

**DOI:** 10.1371/journal.pone.0040646

**Published:** 2012-07-17

**Authors:** Christine Assaiante, Sophie Mallau, Jean-Luc Jouve, Gérard Bollini, Marianne Vaugoyeau

**Affiliations:** 1 Laboratoire de Neurosciences Cognitives, CNRS-Aix-Marseille Université, Centre St Charles, Marseille, France; 2 Service de Chirurgie Orthopédique Infantile, Centre hospitalo-universitaire de la Timone, Marseille, France; The University of Western Ontario, Canada

## Abstract

**Introduction:**

It has been reported that AIS rely much more on ankle proprioception to control the amplitude of the balance control commands as compared to age-matched healthy adolescents. Our hypothesis was that AIS do not neglect proprioceptive information to control posture probably because of their vestibular deficits. We investigated the proprioceptive contribution to postural control in AIS which expresses spinal deformity during a crucial transitional period of ontogenesis.

**Methods:**

10 adolescents with idiopathic scoliosis (AIS) with moderate spinal deformity (10° < Cobb Angle >35°) and 10 control adolescents (CA) had to maintain vertical stance while very slow oscillations in the frontal plane (below the detection threshold of the semicircular canal system) were applied to the support with the eyes open and closed. Postural orientation and segmental stabilisation were analysed at head, shoulder, trunk and pelvis levels.

**Results:**

Scoliosis did not affect vertical orientation control and segmental stabilization strategies. Vision improves postural control in both CA and AIS, which seem more dependent on visual cues than adults.

**Conclusions:**

AIS as CA were unable to control efficiently their postural orientation on the basis of the proprioceptive cues, the only sensory information available in the EC situation, whereas in the same condition healthy young adults present no difficulty to achieve the postural control. This suggests that AIS as CA transitory neglect proprioceptive information to control their posture. These results and previous studies suggest the existence of different afferent pathways for proprioceptive information subserving different parts in sensory integration of postural control. We conclude that the static proprioceptive system is not affected by the idiopathic scoliosis, while the dynamic proprioceptive system would be mainly affected.

## Introduction

Idiopathic scoliosis is a developmental pathology which expresses spinal deformity involving all 3 spatial planes [1], mainly during adolescence. Adolescence is a period of physiological and psychological transition between childhood and adulthood, which is known to involve considerable morphological, structural and functional changes [2], [3].

In static conditions, postural control implies the choice of a given body orientation, generally aligned to the gravity vector, and the maintenance of this posture against the perturbing effects of the gravity force and other external forces. It is well established that the visual, vestibular (otolithic) and somatosensory information provide information for estimation of the verticality. In previous study, we have demonstrated in young healthy subjects that proprioceptive cues are predominant in the control of body orientation rather than visual or vestibular cues for the control of upright body posture [4]. In order to find experimental evidence for this hypothesis, we have excluded visual cues (eyes closed) and canalar vestibular cues. The subjects were standing on a motorised platform that oscillates along the frontal and the sagittal planes. The platform’s movement was so low that it would be below the detection threshold for vestibular semi-circular canal stimuli this condition this information should not contribute to balance control This same paradigm applied to healthy adolescents [5] has shown that adolescents’ postural control is much less efficient than those of adults. Moreover, the use of vision improved their postural control in term of both postural stabilisation and postural orientation. This suggests that vision plays a predominant role in adolescents’ control of body’s orientation and stabilisation, as previously reported in infants during the period of acquisition of the main motor abilities [6].

Herman et al. [7] reported that adolescents with idiopathic scoliosis (AIS) exhibit perceptual impairments, deficits in sensorimotor adaptation and balance control during imposed perturbations of the body. According to these authors, these deficits would be the signature of disorders at higher integrative levels of the central nervous system. A recent study [8] also reports that AIS have difficulty in reweighting sensory inputs following a brief period of proprioceptive deprivation during postural tasks. More precisely, their results suggest that the mechanisms in charge of re-adapting the central drive following tendon vibration may respond more tardily in AIS than controls. Simoneau et al. [9] reported that AIS, compared to control adolescents (CA), rely much more on ankle proprioception to control the amplitude of the balance control commands, despite the availability of vision. On the basis of Simoneau’s studies [8], [9] it seems that AIS do not neglect proprioceptive information to control their posture probably because of their vestibular deficit [10], [11]. Thus, we adopted the working hypothesis that, despite the body scheme disturbances including spinal deformity, AIS will not transiently neglect the information provided by the proprioceptive pathway, as CA do, but in contrast may rely more strongly on proprioceptive cues than on other sensory systems such as vision and vestibular static inputs to control their orientation and stabilise their body.

In order to find experimental evidence for this hypothesis, we exclude in our experiments visual cues (eyes close) and vestibular cues, by keeping frequency and amplitude of the tilt perturbation so low that it would be below the detection threshold for vestibular semi-circular canal stimuli. Under these conditions, the postural control strategies used by the subjects with their eyes closed are mainly based on the use of somesthetic information. Using this paradigm, we have previously shown that CA were unable to achieve correctly the postural control on the basis of the proprioceptive cues alone and we have concluded that healthy adolescents transitory neglect the proprioceptive cues. In AIS, we speculate their postural performances would be better than those of the CA tested in this study, attesting an overuse of proprioceptive cues. In this context, if our working hypothesis is true, then the postural performances of our AIS subjects would be better than those of the CA tested in this study.

## Materials and Methods

### Ethical Statement

This experiment was approved by the local ethical committee i.e CPP Sud-Méditerranée I, therefore it has been performed in accordance with the ethical standards of the Declaration of Helsinki. All the subjects and their parents gave their written informed consent prior to the study.

### Subjects

Ten AIS participated in this experiment (mean age 14 years 6 months, SD+/−1 year 5 months; 9 girls and 1 boy). AIS had previously been screened and diagnosed by a trained paediatric orthopaedic surgeon in the orthopaedic care unit of Marseille’s Timone Hospital. No patient was under active treatment, none of the patient had surgery before, and no patient presented any neurological sign of pathologic importance in clinical examination. The average Cobb angle was 18.3° +/−5° and varied between 10° and 35°. Ten CA (mean age 14 years 4 months, SD +/−1 year 2 months; 8 girls and 2 boys) participated in this study. They did not report any neurological or orthopaedic problem. Subject characteristics are listed in Table 1. Parents and adolescents gave their informed consent prior to the experiment, which obtained the approval of the local ethics committee and has therefore been performed in accordance with the ethical standards of the Declaration of Helsinki.

**Table 1:** Subjects’ Characteristics.

	Patients	Controls
**N**	10	10
**Males/Females**	1/9	2/8
**Age**	Mean 14 years 6 months SD 1 year 5 months	Mean 14 years 4 months, SD 1 year 2 months
		
**Mean Cobb Angles (°) (SD)**	18.3° (5°)	NA
**Scoliosis type**		
**Left lumbar**	3	NA
**Right thoracic**	3	NA
**Thoraco-lumbar**	4	NA

### Task and Surroundings

Subjects stood on a motorised uni-directional rotating platform with their eyes open (EO) or closed (EC). The platform was rotated laterally sinusoidally at 0.01 Hz with amplitude of 10° peak to peak. The subjects were positioned on the platform such as the movement of the platform induced body’s movements mainly along the frontal plan. They had to maintain a vertical posture as steadily as possible, keeping their feet 15 cm apart without flexing their knees. The trial lasted for 106 seconds, including a complete cycle of angular platform movement (100 seconds). The maximum angular accelerations of the platform were for this amplitude and at this frequency well below the vestibular detection threshold, i.e. 0.2°/s^2^ (Henn et al., 1980). Even at this low frequency, AIS and CA were aware that the platform was rotating.

### Data Collection

**Figure 1:**
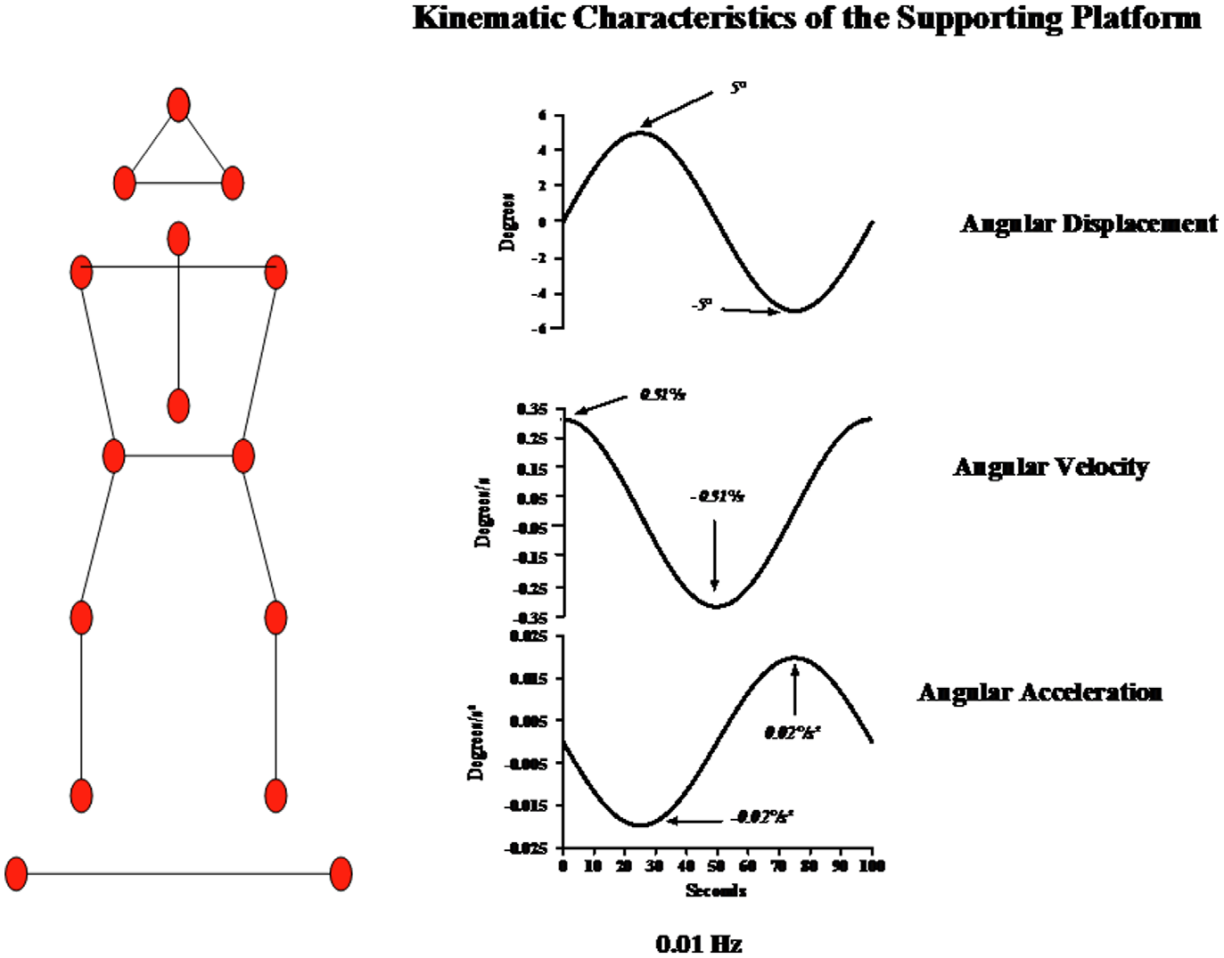
Experimental set up. Left panel: Arrangement of the markers used to measure the effects of the lateral disturbance applied to the supporting platform. The 13 markers were placed symmetrically in pairs on the subject’s back at the following sites: top of the head, mastoid, acromion process, spinal process of C7, on the sacrum, posterior-superior iliac crest, lateral tibial plate, external malleolus. Two supplementary markers were also placed on the supporting platform to measure its lateral movements. Right panel: Characteristics of the supporting platform’s movement at 0.01 Hz The top curve illustrates the angular displacement of the supporting platform. The middle curve illustrates the angular velocity of the supporting platform and the bottom curve illustrates the angular acceleration of the supporting platform. The arrows indicate the peak of inclination, of velocity and of acceleration.

Data collection was performed with the SMART automatic motion analyser (eMotion) working at 120 Hz, using passive body markers. Subjects performed the task facing 6 SMART TV cameras and wearing 13 markers (15 mm in diameter) onto the skin, placed symmetrically on the adolescent’s back at the following sites: top of the head, mastoids, spinal vertebral process C7, acromial process, sacrum, posterior-superior iliac crest, lateral tibial plateau and external malleoli. 2 supplementary markers were placed on the platform to measure its oscillations along the frontal plan, as indicated in figure 1.

### Indexes

#### Analysed segments

The postural stabilization and postural orientation control of head, trunk, shoulder and pelvis were analysed. The head was defined as a vector located between the right and left mastoid markers, the trunk as a vector between markers located on spinal vertebral process C7 and sacrum, the shoulder as a vector between markers located on the right and left acromion and the pelvis as a vector between markers located on the 2 rigth and left posterior-superior iliac crest.

Three trials were run with each subject in each experimental condition. The order of presentation of the experimental conditions was pseudo randomized. Three controlled variables were used to estimate both segmental orientation (sequential mean orientation, angular dispersions) and stabilisation (anchoring index). The controlled variables were averaged in all subjects and all trials in each experimental condition.

#### Sequential orientation

For each subject a reference orientation value was obtained for each body segment during 10 seconds of upright stance on a stable support with the EO. At each trial, the mean value of the absolute angular variation as function of time was calculated for each body segment (head, shoulders, trunk and pelvis in response to lateral oscillations of the platform). This value was calculated during a whole cycle of platform movement. To obtain the *mean orientation* of one segment during one trial, the reference value was subtracted from the mean value of the absolute angular distribution recorded during that trial. Likewise, the *sequential mean orientation* of each body segment was calculated using the same procedure, during each tenth of a cycle (10 s) of platform movement, in order to assess the time course of the segmental orientation process.

#### Angular dispersions

The absolute head, shoulder, trunk and pelvis roll angles were computed during a trial. For each trial, the standard deviations of the absolute angular distributions (noted Sd Abs) were then calculated in order to give an indication of the amplitude of the oscillations at these various anatomical levels.

#### Anchoring Index (AI)

**Figure 2:**
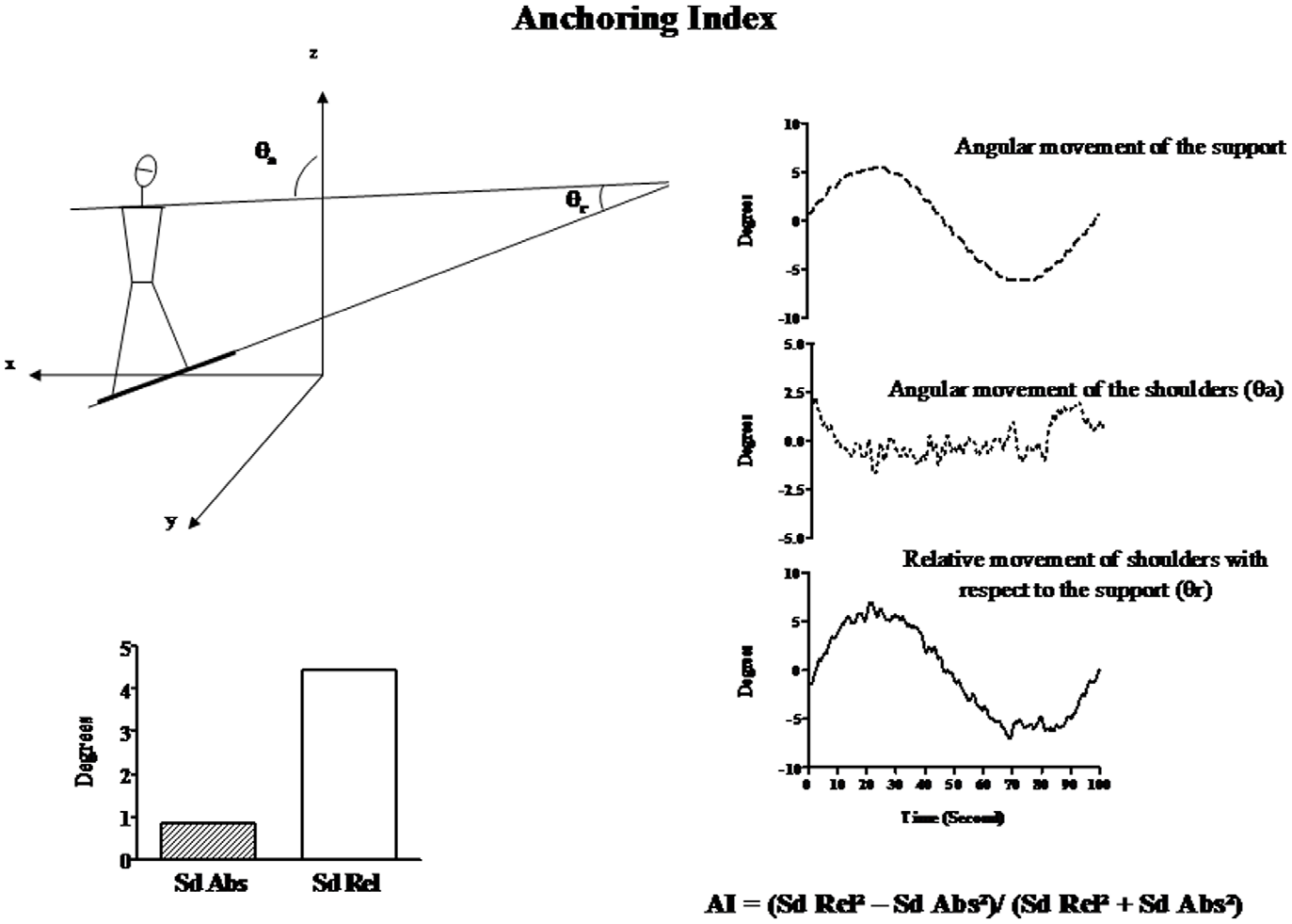
Anchoring index calculation. Left upper panel: Diagram of the shoulder roll angle with respect to the external axis, θ_a_, and with respect to the supporting platform, θ_r_. With x: lateral axis, y sagittal axis and z vertical axis. Right panel: angular roll displacement of the supporting platform (upper trace), the absolute angular displacement of the shoulders (middle trace) and the relative angular movement of the shoulders with respect to the supporting platform (lower trace). Left lower panel: Diagram of the absolute (Sd Abs) and relative (Sd Rel) roll dispersions of the shoulders, according to the definition of the AI (AI). In this example, AI is positive, which means that the shoulders are stabilised in space independently of platform movements.

Segmental stabilization was defined in terms of the global AI calculated during the whole cycle of perturbation [5], [4], [12], [13], [14], [15], [16], [17], [18]. The segmental AI was used to compare the stabilization of a given segment with respect to both an external reference value and the moving platform. AI was calculated for each trial as follows (figure 2).

AI  =  (Sd Rel^2^ – Sd Abs^2^)/(Sd Rel^2^ + Sd Abs^2^) where Sd Abs is the standard deviation of the angular distribution about the roll of the segment under investigation with respect to the absolute allocentric reference (absolute vertical direction) value and Sd Rel is the corresponding standard deviation of the angular distribution with respect to the moving platform. A positive AI indicates a better segmental stabilization along the absolute vertical axis than in response to the moving platform, whereas a negative value indicates a better segmental stabilization on the platform than on the external absolute axis.

### Statistical Analysis

Three trials for each subject in each experimental condition were analysed. The medians of the three trials in each experimental condition were calculated and used for statistical analysis. The statistics given in text and figures are medians and interquartiles. Differences between CA and AIS were tested with a Mann–Whitney *U* test. The effects of vision were analyzed by comparing the performances of each group with and without vision, using Wilcoxon's signed rank test for within-subject comparisons. AI were compared to zero, using the Wilcoxon’s test to determine whether single-sample procedure (against the null hypothesis). Since these indices were in the −1 to +1 range, a z transform was used to convert the values into an unbiased Gaussian distribution. Differences with a p value <0.05 were taken to be statistically significant.

## Results

### Postural Orientation

#### Sequential orientation

**Figure 3:**
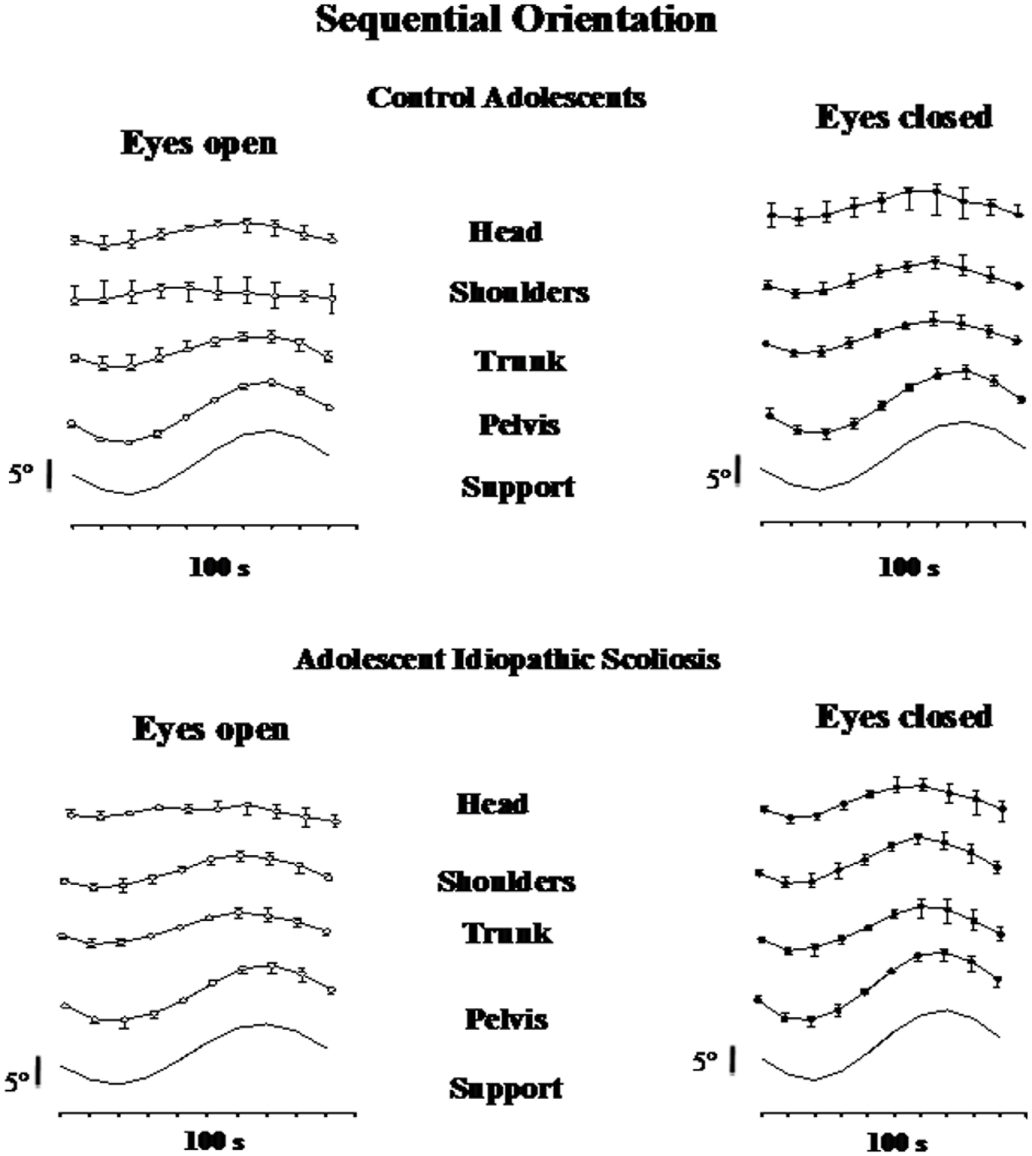
Sequential orientations. Median and quartiles of sequential orientation of head, shoulders, trunk, pelvis and support (top to down) in controls (top panels) and adolescent’s idiopathic scoliosis subjects (down panels) with eyes open (left panel) and eyes closed (right panel).

The sequential orientation of each considered segment, with and without vision, and the median, 1^st^ and 3^rd^ quartiles values of angular dispersions of the pelvis, trunk, shoulders and head, for both groups of subjects are shown in figure 3.

The qualitative analysis of figure 3 shows that in both groups, head, shoulders and trunk sequential orientation showed few variations with time, which showed that the oscillations induced in the various anatomical segments, were efficiently attenuated. However, without vision, CA as well as AIS tended to slightly follow the movement of the platform, especially at trunk level. At the pelvis level, in both groups of subjects and whatever the visual condition, the pelvis followed the movements of the supporting platform.

#### Angular dispersions

**Figure 4:**
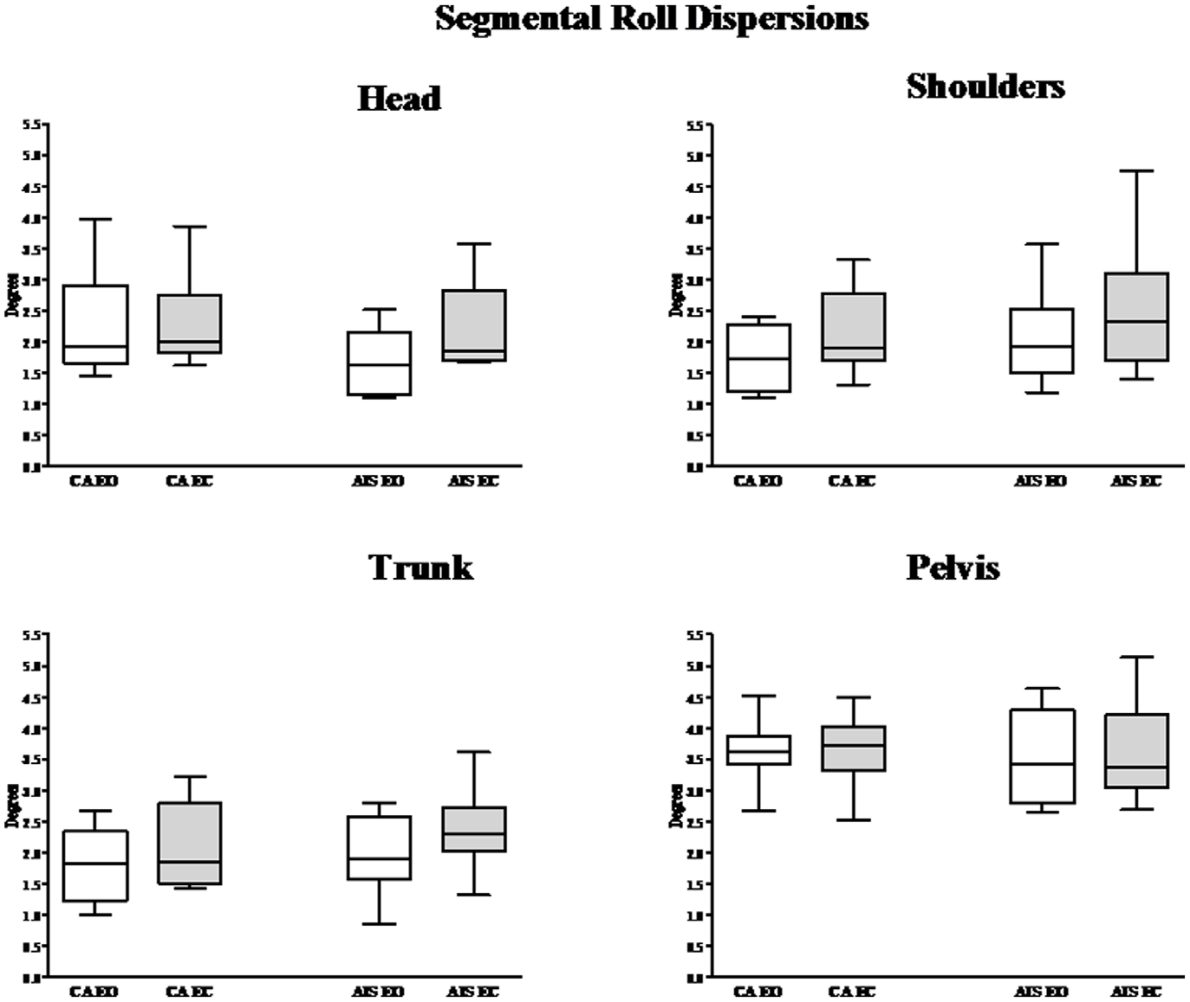
Segmental angular dispersions. Median and quartile of the segmental roll dispersion of head, the shoulders, the trunk and the pelvis in control subjects and adolescents idiopathic scoliosis subjects with eyes opened (EO, white) and eyes closed (EC: black).

The segmental roll dispersion of each considered segment, with and without vision, and the median, 1^st^ and 3^rd^ quartiles values of angular dispersions of the pelvis, trunk, shoulders and head, for both groups of subjects are shown in figure 4.

The attenuation of the oscillations induced in the anatomical segments was assessed in terms of the segmental roll dispersions. When the platform oscillated in the frontal plane, with and without vision, no statistically significant difference in the angular head, shoulders, and trunk and pelvis dispersion was observed between CA and AIS.

CA showed greater shoulders and trunk angular dispersions without vision than with vision (and W = 53, p<0.001, and W = 47, p<0.01 at shoulders and trunk level, respectively). A similar tendency was observed in AIS at head and trunk level (W = 55, p<0.01 and W = 34, p<0.05, at head and trunk level respectively). No significant effect of vision was revealed at the pelvis levels for CA as well as for AIS.

#### Postural stabilization

**Figure 5:**
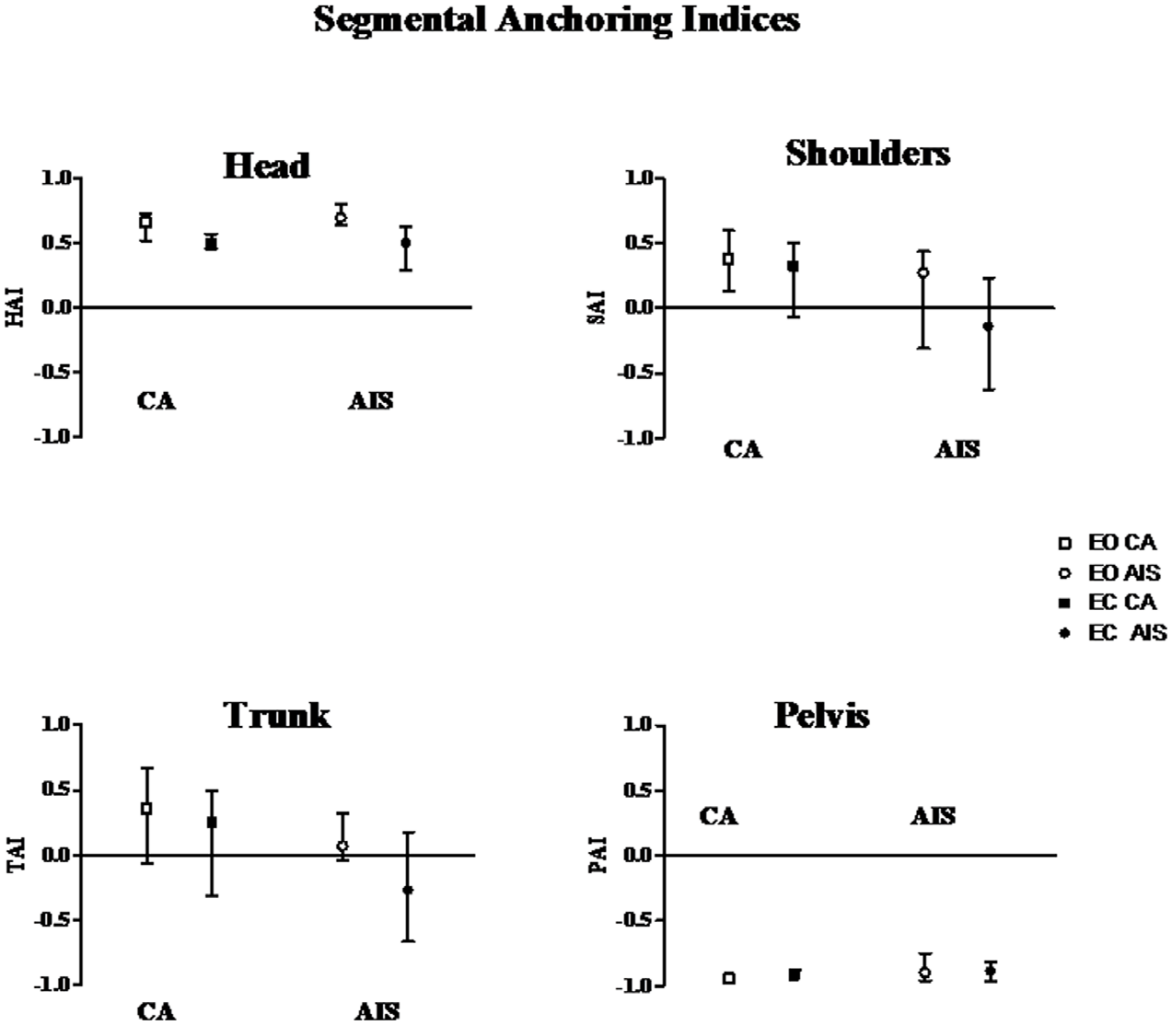
Segmental anchoring index. Median and quartile of AI of the head (HAI), the shoulders (SAI), the trunk (TAI) and the pelvis (PAI), in control subjects (CS: square) and adolescents idiopathic scoliosis subjects (AIS: circle) with eyes opened (EO, white) and eyes closed (EC: black).

The median, 1^st^ and 3^rd^ quartile values, of pelvis, trunk, shoulders and head AI, with and without vision for each group of subject are shown in figure 5.

With and without vision, no statistically significant difference in the head, shoulders, and trunk and pelvis AI values was observed between CA and AIS.

The head AI (HAI) values were positive whatever the vision condition in CA (W = 36, p<0.01; W = 49, p<0.01; with vision and without vision respectively) and in AIS (W = 36, p<0.01; W = 32; p<0.001 with vision and without vision respectively). The comparison of the HAI with and without vision revealed a significant decrease in CA (W = −43; p<0.01) as well in AIS (W = −41 p<0.05) in absence of vision only.

The shoulders AI (SAI) values were significantly positive with vision for CA (W = 19, p; p<0.05) but no significantly different from 0 for the AIS. Without vision, the SAI of CA and AIS were not significantly different from zero. The comparison of the subjects performances with and without vision revealed a significant decrease of the SAI without vision in both group of subjects (W = −30; p<0.05 and W = −53; p<0.001 for CA and AIS respectively).

The trunk AI (TAI) were not significantly different from zero for both groups of subjects and whatever the visual condition. The comparison of the subjects performances with and without vision revealed a significant decrease of the TAI without vision in both group of subjects (W = −45; p<0.05 and W = −43; p<0.01 for CA and AIS respectively).

The pelvis AI (PAI) were significantly negative in CA (W = −55; p<0.001 and W = −55; p<0.001 with and without vision respectively) and in AIS (W = −55; p<0.001 and W = −55; p<0.001 with and without vision respectively) whatever the visual condition. The comparison of the subjects’ performances with and without vision did not reveal any effect of vision nor in CA neither in AIS.

## Discussion

The aim of the present study was to investigate the proprioceptive contribution to postural control in AIS. In other words, we would like to study how developmental process interferes with pathology which expresses spinal deformity during adolescence that is a crucial transitional period of ontogenesis. The way to assess the ability of AIS to transform available sensory inputs into appropriate motor commands was to manipulate sensory information and quantify its effect on balance control by means of an original procedure that consists in applying very slow oscillations on the support on which the subject is standing.

The results show that 1) the AIS and CA performances were affected in terms of both the postural orientation and stabilization components, 2) the use of vision improved postural performances in both groups of adolescents.

### Moderate Spinal Deformity did not Affect Postural Control

It emerges from this study that moderate spinal deformity did not affect vertical orientation control and segmental stabilization strategies, the performances of AIS were the same than those of CA. Concerning the body orientation, the angular dispersions decreased from the pelvis to the head indicating that support oscillations were more damped at head and shoulders levels in both groups. As showed in previous studies, with this protocol, in children and in healthy adolescent [5], [15], no attenuation of the oscillatory pattern induced by the platform was observed at the pelvis in AIS as CA who used the foot support as their reference frame. No preferential strategy to stabilize the upper segments (shoulders and trunk) was systematically adopted by both groups. By contrast, AIS as CA adopted the head stabilization in space strategy in response to slow oscillations of the support. A difference between CA and AIS in the head stabilization strategy was previously reported during locomotor tasks on narrow supports [14]. Probably the specificities of the slow oscillations protocol could explain this difference. Indeed, the slow oscillations of the support include a maximum tilt of 10° that does not represent a major balance difficulty as the walk on narrow supports can be. During quiet stance, Herman et al. [7] also reported no difference in body sway between CA and AIS.

### Prevalence of Visual Contribution to Postural Control in AIS and CA

By contrast with young adults [4] the use of visual cues was found to improve the adolescents’ postural performances in terms of the orientation and stabilisation of the upper body segments in both CA and AIS. Similar effect were highlighted in several developmental studies [19], [5], [15], [20] showing that children’ and adolescents’ postural performances decreased in the absence of vision. Ferber-Viart and colleagues [21] concluded that in postural control, somatosensory inputs are primary in adults while vision predominates in children. In our study, without vision the postural impairment was not larger in AIS. This result contrasts with previous studies in AIS indicating that when visual feedback was removed, body sway increased significantly more in AIS than in CA [7], [22]. Probably that this potential difference between CA and AIS, reported in the literature, was masked in our study because of the increased visual contribution in CA due to their transitory neglect proprioceptive information to control their posture in response to very slow oscillations of the support [5].

### AIS as CA Transitory Neglect Proprioceptive Information

We have previously shown [4], [12] that adults can maintain vertical stance on the basis of proprioceptive information alone. Indeed, using the same experimental paradigm on healthy young adult, we have established that in the absence of visual information, oscillatory perturbations applied to the foot support below the vestibular perception threshold did not affect the subjects’ ability to control vertical posture in term of both stabilization and orientation [4], [12]. The present study shows that the performances of both groups of adolescents are much less efficient than those of adults when no visual cues are available. These results suggest that both groups of adolescents were not able to use the only one proprioceptive information available to improve their postural control. These results suggest that a transient period of proprioceptive neglect occurs in sensory integration of postural control during adolescence in CA and in AIS. In our study, it seems that developmental effect is dominant with respect to pathologic effect. By contrast with our initiate speculation, AIS as CA transitory neglect proprioceptive information to control in response to very slow oscillations of the support.

### Proprioception Involves Several Functions

Nevertheless, several paradigm assessing proprioceptive contribution to postural control in AIS reported sensory integration problem, proprioceptive disorders translated by larger body sway in AIS than age-matched healthy controls [23], [7], [8], [9]. Probably that these apparent opposite results between our study and the literature suggest the existence of different afferent pathways for proprioceptive information subserving different parts of the motor program of postural control [24]. Indeed, as it has been proposed by Goldscheider [25], proprioception involves several functions: movement sense (assessing by short duration tendon vibrations application), that is the ability to detect direction, amplitude and speed of movements, and position sense (assessing with slow passive movement), that is the ability to compare the final and initial positions, in order to recognise whether a movement has been performed.

Positron emission topography (PET) studies have shown that these two proprioceptive sub-systems are underlined by different patterns of brain activation [26]. More precisely, brain activity were recorder using PET in 4 four conditions (1) passive flexion-extension movement of the left forearm; (2) induced illusions of movements similar to the real passive movement; (3) alternating vibration of biceps and triceps tendons without induced kinesthetic illusions and (4) rest condition (RE). The results of this study revealed different patterns of cortex activation. The comparison of the brain activities during passive movement with those obtained during vibration revealed activation of following areas the primary motor and somatosensory area, the SMA and the supplementary somatosensory area. In conditions where passive movements and illusory movements were contrasted with rest, some temporal areas (primary and associative auditory cortex) were activated, as well as secondary somatosensory cortex. This study shows that different proprioceptive inputs are associated with differently located activation patterns of the cortex. Goodwin *et al.* [27] found that the kinaesthetic illusion from vibration was predominantly one of movement, although there was a perceived change in limb position during and at the end of vibration. It has been demonstrated that if movements are made progressively more slowly, what started out as a movement sensation eventually blends into a sense of changed position [28], [29]. So, the present paradigme where the supporting platform’s movements were very slow, investigate preferentially the position sense, i.e the static proprioception.

Taken into account the literature and our results, we conclude that the static proprioceptive system, as assessed from our protocol, would be not affected by the idiopathic scoliosis, while the dynamic proprioceptive system would be mainly affected. In further investigations, it would be interesting to determine if kinaesthetic illusions elicited by artificially manipulating the proprioceptive channel through tendon vibration are impaired in AIS as compared with CA. Tendon vibration stimulation provides a classical relevant mean of challenging the dynamic proprioceptive system in postural task. In this line, we speculate a specific involvement of the dynamic proprioceptive cues causing a specific balance postural deficit in moderate spinal deformity while static process may be preserved or damaged with respect to the severity of the spinal deformity.
